# Distribution and Morphological Characteristics of Oligodendrocytes in Selected Areas of the Brain of Male and Female Red Kangaroos (*Macropus rufus*)

**DOI:** 10.3390/brainsci12081035

**Published:** 2022-08-04

**Authors:** Agata Wawrzyniak, Krzysztof Balawender, Roman Lalak, Maciej Przemysław Golan, Konrad Wróbel, Dariusz Boroń, Rafał Staszkiewicz, Beniamin Oskar Grabarek

**Affiliations:** 1 Department of Morphological Sciences, College of Medical Sciences, Institute of Medical Sciences, University of Rzeszow, 35-315 Rzeszow, Poland; 2 Department of Animal Anatomy and Histology, University of Life Sciences in Lublin, 20-400 Lublin, Poland; 3 Laboratory of Molecular Oncology and Innovative Therapies, Military Institute of Medicine in Warsaw, 04-141 Warsaw, Poland; 4 Department of Histology, Cytophysiology and Embryology, Faculty of Medicine in Zabrze, Academy of Silesia in Katowice, 41-800 Zabrze, Poland; 5 Department of Gynaecology and Obstetrics, Faculty of Medicine in Zabrze, Academy of Silesia in Katowice, 41-800 Zabrze, Poland; 6 Department of Neurosurgery, 5th Military Clinical Hospital with the SP ZOZ Polyclinic in Krakow, 30-901 Krakow, Poland; 7 GynCentrum, Laboratory of Molecular Biology and Virology, 40-851 Katowice, Poland

**Keywords:** red kangaroos (*Macropus rufus*), oligodendrocytes (OLGs), hippocampus (Hip), corpus callosum (CC), histochemical studies

## Abstract

This study was carried out on six adult red kangaroos of both sexes. To determine the location of the oligodendrocytes (OLGs) of the hippocampus (Hip) and corpus callosum (CC), the method of impregnation of the neuroglia with silver salts was applied. The iron distribution in the OLGs was determined by the histochemical method. The Nissl method was used to determine the location of the brain structure and to analyze the number of OLGs. In the Hip, these cells are located one beside another, mainly in blood vessels and neurons; in the neocortex (NC), they are located in layers I–VI; and in the CC, they are arranged in characteristic rows and accompany both nerve fibers and blood vessels. The analysis of the results obtained by the chosen methods in the Hip, NC, and CC in males and females did not show statistically significant differences in the distribution and location of the red kangaroo OLGs. The involvement of these cells is a physiological process that proceeds in a similar manner throughout the life of individuals and actively influences the metabolism of neurons and myelin.

## 1. Introduction

Pío del Río Hortega first classified OLGs in regard to their position: perineuronal and interfascicular. However, the next classification was based on his analysis of the number and branching patterns of the processes. He found four patterns that, in his opinion, described the branching patterns of the processes of all OLGs. The first two types of OLGs had branches scattering in many directions; the other two forms dispatched processes parallel to the adjacent myelin sheaths and therefore assumed a much more linear overall pattern. The mature OLG is a small cell with a marked density of both the nucleus and the cytoplasm [1]. These cells have a round or oval nucleus with substantial amounts of heterochromatin that occupy the nuclear periphery and contribute to its dense appearance. The density of the oligodendrocyte cytoplasm is believed to be caused by densely staining material located in the cytosol between the organelles [2]. There are no unique organelles within the OLGs’ cytoplasm. The rough endoplasmic reticulum is well developed, and the cisternae tend to be flattened. The Golgi apparatus of the OLGs contains cisternae that often appear distended, but the mitochondria are relatively small. Some mature oligodendrocytes contain distinct lamellar bodies of unknown function that appear to increase with age [2]. Mature OLGs have also been found to contain numerous microperoxisomes within the cytoplasm. These peroxisomes most likely play a role in lipid metabolism related to myelin synthesis or maintenance. Their cytoplasm lacks cytoplasmic fibrils, glycogen granules, and frequent dense bodies [1].

More recently, the use of antibodies for specific myelin components has allowed direct demonstration of the continuity between the oligodendrocyte cell body and myelin sheath formation. Staining slices of the myelin basic protein (MBP) can demonstrate the continuity of the OLGs adjacent to the myelin segments under the light microscope. Mori and Leblond [2] noticed that the various forms of oligodendrocytes found in electron micrographs of the adolescent rat corpus callosum could be arranged in a continuous spectrum and identified them as three classes of OLGs. They were described in detail and called “light”, “medium”, and “dark” OLGs [3]. Light OLG was characterized by a large euchromatic nucleus with few chromatin masses filled with organelles and free ribosomes. It contains a small rough endoplasmic reticulum and a variable Golgi apparatus. The medium OLG was smaller and electron-denser than the light one. Its nucleus was smaller with more clumps of chromatin, and the cytoplasm decreased in volume, with fewer free ribosomes. Still, it contained a more prominent Golgi apparatus and longer and more regularly stacked cisternae of the rough endoplasmic reticulum. This type had fewer processes than the light variety. The dark OLG had a small electron-dense nucleus and an electron-dense cytoplasm. It contained stacks of rough endoplasmic reticulum cisternae and a somewhat smaller Golgi apparatus also with lucent cisternae.

The relative percentages of the light, medium, and dark oligodendrocytes found by Mori and Leblond [2] in their study of young rats were 6, 25, and 40% of the total glial population, respectively. The light OLG was considered the myelinating cell, but the dark OLG was a mature cell engaged in myelin maintenance. At early ages, light and medium varieties together comprised 30 to 60% of the total OLG population, but the number of cells in these two categories decreased in mature adults, so the dark variety proved to make up more than 90% of the total oligodendrocytes. Mori and Leblond [2] supposed that the light OLG was capable of further division while maintaining its processes and that the light OLG stock was partly supplemented by this division [1]. Oligodendrocytes are the myelinating cells of the CNS. They must undergo a complex and precisely timed program of migration, differentiation, proliferation, and myelination to finally produce the insulating sheath of axons. Due to this complex differentiation program and due to their unique metabolism/physiology, OLGs are among the most vulnerable cells in the CNS. The time interval available for the onset of myelination appears to be very short, in the range of 12–18 h, during which OLGs must wrap layer after layer of plasma membrane around multiple axons [4]. The iron distribution in the white matter, although unique to oligodendrocytes, is not homogeneous but rather occurs in patches.

In the developing brain, iron is first detected in the OLGs proximal to blood vessels [5]. During the second postnatal week, the distribution of iron-positive oligodendrocytes colocalizes with the myelinated fibers. This is evidence of the functional relationship between iron accumulation and myelin production. Moreover, the iron acquisition by OLGs at the peak of myelination is probably linked to their energy metabolism [6]. Several highly specific proteins are found only in OLGs and myelin. These major proteins are myelin basic protein (MBP), proteolipid protein (PLP), myelin-associated glycoprotein (MAG), or myelin oligodendrocyte glycoprotein (MOG). These proteins are exclusively produced by OLGs in the CNS and thus serve as excellent markers for myelinating cells [1]. In addition to neurons, the normal brain is constituted by glial cells, astrocytes, OLGs, and microglial cells, but also how radical the phenotype of glial cells is in all brain injuries and diseases [4]. Many diseases of the nervous system involve myelin. Multiple sclerosis (MS) is one of the most common neurological diseases. It involves demyelination due to an autoimmune attack on myelin and OLGs. It seems that in MS, there is initial remyelination due to the generation of new oligodendrocytes and new myelin; at some point in the disease, this repair process fails. Many OLGs are lost in brain trauma and spinal cord injury either directly or indirectly due to axon injury and degeneration [7]. Regions that contain exclusively unmyelinated nerve fibers, such as the retina (in many species) or the molecular layer of the cerebellum, do not have OLGs [8].

The neocortex (NC) is the part of the mammalian brain involved in higher-order brain functions such as cognition, sensory perception, generation of motor commands, spatial reasoning, and language [9]. The neocortex represents the large majority of the outer layer of the cerebrum, with the allocortex making up the rest [9]. The neocortex represents the grey matter, which consists of neuronal cell bodies and unmyelinated fibers surrounding the more profound white matter (myelinated axons) in the cerebrum. In primates, the NC has six layers labeled from the outermost inward, I to VI, and contains about 10 to 14 billion neurons. Each cortical layer contains different neuronal shapes, sizes, and densities, as well as different organizations of nerve fibers [10]. In the human brain, white matter volume decreases by 28% [11] and the length of subcortical myelinated fibers decreases by 45% from the age of 20 to the age of 80 [12]. There are about 5000 to 10,000 synapses per neuron in the neocortex, resulting in approximately 150,000 billion in the NC [13]. To date, very little is known about the number of NC and OLGs as a function of age. A non-stereological study on neocortical cell counts has reported a loss of neuronal cells with age but an increasing number of glial cells. In the human brain, the number of OLGs correlates with both the volume of the neocortex and the total number of neurons in the neocortex. As mentioned above, the length of myelinated neuronal fibers decreases by approximately 45% from the age of 20 to 80 years [12], whereas the number of neurons decreases by approximately 10% in adult life [11]. The number of OLGs decreases by 27% in adult life from the age of 20 to 90, proving there is an interaction between neuronal activity and the number of OLGs [14].

The CC is the largest commissure in the central nervous system (CNS) and provides the main white matter link between the hemispheres, connecting the homotopic and heterotopic cortical regions and is easily delineated anatomically [15]. The CC represents a white matter tract that is modulated by experience throughout life. This white matter tract is modulated by experiences and therefore represents a good model for studying myelination. The number of oligodendrocytes in CC is established in childhood and remains stable after that. Acquired damage or agenesis of CC impairs bimanual motor performance, especially for tasks that require coordinated asynchronous bilateral movements. Strong interhemispheric connections exist between the motor areas and between the CC and the cortex [15]. In the CC, the number of OLGs was the highest in the youngest individuals. The number then dropped during early childhood to approach constant numbers at about five years of age. Shortly after birth, in the human brain in the CC, there is a limited number of mature OLGs, but it increases in the perinatal period. At about five years of age, the final number of OLGs reaches a stable value of about 88%. The stereological data in the human brain in the CC indicate that about 98.5% of the final number of OLGs is obtained at about nine years of age. After this period, the number of OLGs remains mostly stable throughout the rest of the lifespan [16]. The turnover of OLGs contributes minimally to the modulation of myelin in human white matter, which can be carried out by mature OLGs, which may facilitate rapid neural plasticity. The proliferation of OLG and the initiation of myelination were suggested to be regulated by neural activity. Myelinating OLGs continue to be generated in adulthood at a substantial rate in rodents, but it is less clear to what extent mature oligodendrocytes can modulate their myelination [17]. Most functional changes in the CNS with normal aging are associated with a decrease in learning and memory. 

It is well accepted that the Hip is a very important region because it is connected to other brain regions related to learning and memory [18]. Both in animals and humans, the severity of cognitive disorders has been positively correlated with the extent of functional and structural modifications that occur in the Hip. The Hip is considered to be the most vulnerable region to changes in normal Hb levels and neurodegenerative diseases [19]. Normal aging and degenerative processes in the Hip are accompanied by changes in the number and function of neurons and glia, their volumes, and various factors such as neurotransmitters and hormones [20,21]. The Hip is one of the oldest brain regions, phylogenetically. It consists of two major parts, the Hip proper and the dentate gyrus (DG). The Hip proper contains four subregions (CA1–CA4), and each subregion consists of four distinct layers: stratum oriens (SO), stratum pyramidale (SP), stratum radiate (SR), and stratum lacunosum-moleculare (SLM). The main excitatory output neurons, referred to as pyramidal neurons, are located in the SP [22]. The Hip is involved in various physiological functions such as olfaction, arousal, cognition, learning, and memory [23]. Although the controversy over the exact functional role of the Hip subregion is ongoing, the CA1 region appears to be related to the association and completion of temporal patterns as well as intermediate-term memory.

Furthermore, the CA1 sector of the hippocampus is one of the initial brain regions that shows pathology and neuronal loss in Alzheimer’s disease. The CA3 region mediates the processes related to spatial pattern association and completion, as well as short-term memory. The DG is involved in the metric spatial representation and the spatial pattern separation [23,24,25,26,27,28,29]. Most studies today on brain aging have focused on estimates of the total number of neurons in defined brain regions. In contrast, fewer studies have investigated quantitative and functional reports in OLGs, mainly in rodents, non-human primates, and human brains. As the literature lacks data on OLGs in kangaroos, this article is focused on comparative analysis: the location and morphology of OLGs, cellular and regional distribution of iron, the density of OLGs, and level of protein expression of MBP and its molecular weight in the NC, Hip, and CC in adult male and female kangaroos.

## 2. Materials and Methods

### 2.1. Animals

The approval was granted by the 2nd Local Ethical Committee in Lublin No. 78/2014.

This study was performed in accordance with the “Guide for the care and use of laboratory animals” approved by the National Research Council and our local law regulations. The animals came from zoological gardens in Warsaw (*n* = 4) and Zamość (*n* =2), Poland. They died a natural death. The study was carried out on six adult animals of both sexes (three females and three males).

Kangaroos were provided with unlimited access to the habitat, as well as to a roofed external room (250 m^2^) and an internal room (15 m^2^). During the working hours of ZOO employees, i.e., from 8:00 a.m. up to 6:00 p.m., the habitat was monitored by trained personnel who monitored animal welfare twice a day and instructed zoo visitors about the applicable rules. The food of the kangaroos included fresh grass as well as green vegetation (mulberry and eucalyptus), which they received once a day. Three times a week, the kangaroos received an additional 1.8 kg of chopped vegetables and fruits, such as carrots, sweet potatoes, and apples.

Additionally, each kangaroo received 1 kg of Mazuri Macropod Low Starch pellet feed (No. 0038665, PMI Nutrition International, LLC, St. Louis, MO, USA) and 3–4 flakes of soft hay for the group, reduced to 1–2 petals in the summer.

During their stay in the zoo, no abnormalities in the development of kangaroos, or illnesses, were noted.

Kangaroos died of natural causes at the age of 17.3 ± 1.10 (male kangaroos: 16.4 ± 1.61; female kangaroos: 17.12 ± 0.98; *p* > 0.05). The average life expectancy of kangaroos in the wild is about 20 years. The organs were collected for examination during the routine necropsy of the animal.

### 2.2. Sectioning and Histology

Material for the study was collected over several years between 2014 and 2021. After the death of the animals, the brains were removed from the skulls (as soon as possible after the animal’s death, maximum 15 min) and weighed using a digital scale. The weight of the male’s brain was 72 ± 8.10 g, and for the female’s brain, it was 58 ± 4.10 g, and the weight was statistically significantly different (*p* < 0.05). After weighing, the brains were placed in 4% buffered formaldehyde at pH 7.4 (Sigma Aldrich, St. Louis, MO, USA). After fixation, the brain was dehydrated in ethyl alcohol and embedded in the paraffin blocks. The paraffin technique is a standard and very frequently used histological technique. It is a very useful method for research because material fixed and embedded in paraffin blocks can be kept for a very long time. This does not change the structure and as an additional advantage, paraffin sections can be used in various histological stains.

All the procedures used were performed manually and in the same way. The brain samples were cut into smaller parts, and each part was individually dehydrated and embedded in paraffin. Brains were serially sectioned with a Leica RM2255 microtome (Leica, Munich, Germany) into frontal sections, which were then randomly selected and analyzed cytoarchitectonically. Each brain area was defined according to its anatomical location and morphometrically using a computer image analysis system equipped with the LAS V4.7 software package with the Montage module (Leica Application Suite, Leica, Munich, Germany) and Leica DM6000B light microscope (Leica Application Suite, Leica, Munich, Germany).

Specific methods were selected for histological analysis of OLGs, and depending on the histological procedures, different slice thicknesses were required. The Nissl method was used as the initial method to observe the cytoarchitectonics, location, and distribution and measure the density of OLGs. The other methods were used only for its confirmation, although they were used specifically to demonstrate the presence of OLGs. For silver impregnation and iron detection methods, which by design require suitably thick slides, the slides were cut to a thickness of 20 um. For immunofluorescence staining, they were cut to 5 µm thickness.

The same slide-cutting procedure was used for each method. Every third cut slide was selected for staining. It should be assumed that for each stain, the sections originated from the same plane in all compared animals.

### 2.3. Nissl Staining of Oligodendrocytes

The 20 μm thick sections were dewaxed in three changes of fresh xylene, rehydrated in an alcohol series of decreasing concentration, and stained (0.5 g/100 mL) with a solution of Cresyl violet (Merck, Darmstadt, Germany) in distilled water (ratio 1:4) at 37 °C. Next, the sections were differentiated with 96% alcohol until a relatively bright background was obtained. In the next stage, the sections were dehydrated by passing them through a range of alcohols and mounted with DPX (Merck, Darmstadt, Germany).

### 2.4. Morphological Criteria to Differentiate between Glial Cells and Neurons

**Figure 1:**
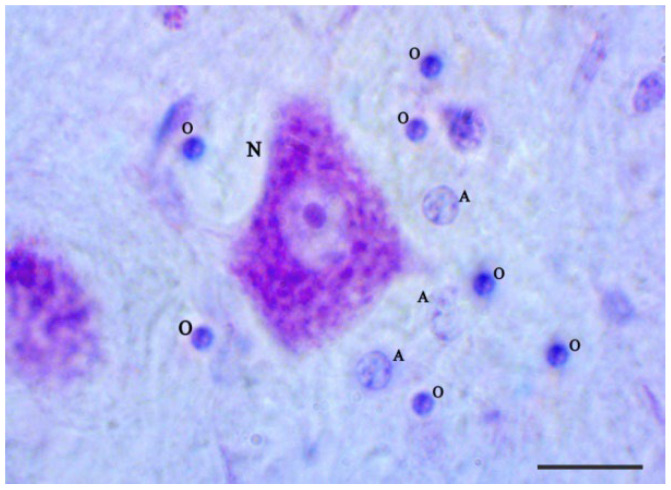
Photomicrographs of the Nissl-stained section are presented in high magnification. The scale bar represents 10 µm. In the Nissl-stained image: N = neuron, O = OLG, A = astrocyte. At higher magnification, note the presence of OLGs in the vicinity of the neurons and astrocytes in the brain. Astrocytes can be distinguished because of a characteristic pale nucleolus.

The application of Nissl bodies allowed us to determine the types of cells in the investigated areas of the brain based on morphological characteristics. Compared to other glial cells, OLGs have a small round or ovoid nuclei with dense, compact chromatin and dark cytoplasm. OLGs are usually arranged in pairs, in groups, or in close proximity to neurons and blood vessels. In contrast to OLGs, astrocytes have round, pale stained nuclei with heterochromatin located mainly in the nuclear cytoplasm shell and are relatively clear [3] (Figure 1).

### 2.5. Quantification

The density measurements of OLGs were made using the Nissl-stained sections (the procedure described above). All measurements were performed, in order to ensure objectivity, under blind conditions by two observers taking the measurements of the experimental sections under the same conditions in each experiment. The cortex layers were identified by their location between the layers that contain characteristic neurons and adjacent white matter. Within the formation of the hippocampal, the posterior part and the part that extends from the lateral geniculate nucleus to the level of the splenium of the CC were analyzed. Images of all structures were taken from four layers of the hippocampus proper: SO, SP, SR, and SLM.

According to the literature [30], CA2 and CA3 were lumped together because these small regions are difficult to separate at the microscopic level due to histological criteria. The NC, Hip (CA1-CA4), and CC (genu, body, and the splenium) were defined according to the atlas of Paxinos and Watson [31]. To estimate the density of OLGs in the NC, Hip, and CC, a stereological approach of systematic random sampling and the unbiased optical fractionator method were applied [30]. Using an optical dissector probe, it is possible to sample isolated particles, in this case, OLGs, with a uniform probability in the three-dimensional space, regardless of the size, shape, or orientation of the tissue. Briefly, the area of interest was delineated in low magnification (2.5×) using the cursor. Anatomical boundaries were designated in the areas examined. A meander sampling function of the LAS4.7 Montage module (Leica Application Suite, Munich, Germany) was used to walk the delineated area with a chosen counting frame. Then, a 100× oil-immersion objective with a numerical aperture of 1.40 was moved into place, and the appropriate counting frame was superimposed on the screen. The desired horizontal and vertical step lengths, assisted by a highly precise servo-controlled motorized microscopy stage, were dimensioned for the appropriate distance (=600 μm (× step) × 600 μm (y step)) between the counting frames (280 μm^2^). An optical dissector probe counted the cells in the space with a height of 15 µm, of the total thickness of the section (26–29 μm). The thickness of each section was measured at three randomly chosen places to obtain the mean value of the thickness of the section. Between 190 and 200 cells were counted per whole structure. This procedure ensured the selection of a systematic random sample of sections, in which all parts of the NC, Hip, and CC had equal probabilities of being presented [30]. The numerical density of the OLGs was estimated as the number of OLGs in 1 mm^3^.

### 2.6. Klüver–Barrera Staining

Klüver–Barrera staining is a method of staining myelinated fibers and nerve cells. When staining tissues, Luxol Fast Blue dye and Cresyl violet are used, which stain the myelin and Nissl bodies, respectively.

The 10 µm thick sections were dewaxed, rehydrated using alcohol series, and washed with 0.01 M PBS (pH 7.4). Then, they were placed overnight in 0.1% Luxol Fast Blue solution in alcohol (Merck, Darmstadt, Germany) at 56 °C and washed twice with a 95% ethanol solution. The operation was repeated until the appropriate degree of color was obtained. In the next step, the sections were stained by the Nissl method according to the above procedure. After staining was complete, the sections were dehydrated in a series of alcohols, immersed in xylene, and sealed with a synthetic medium (Leica Application Suite, Munich, Germany). The obtained sections were compared and analyzed for the correctness of myelination and the absence of demyelinating and dysmyelinating lesions according to Klüver’s recommendation [32].

### 2.7. Impregnation of Nervous Tissue with Silver Salts

The paraffin blocks were cut into 20 µm thicknesses using a Leica RM2255 microtome (Leica Application Suite, Germany). The slides were deparaffinized in three changes of fresh xylene and rehydrated in a series of decreasing concentrations of alcohol and water. After hydration, the sections were dried at room temperature. Next, they were placed on slides and transferred to a 5% water solution of potassium silver cyanide for 24 h at room temperature. The sections were rinsed with distilled water, and an ammonia–pyridine solution was prepared according to Ogawa et al. [33]. After filtering the precipitate, three drops of pyridine were added to the obtained solution. The sections were then reduced with 10% formalin, rinsed with distilled water, and fixed in 5% sodium thiosulfate. In the next stage, the sections were dehydrated by passing them through a range of alcohols and mounted with DPX (Merck, Darmstadt, Germany).

### 2.8. Detecting the Presence of Iron Ions by Histochemical Method

Iron detection histochemical staining was performed using the Perls Prussian Blue Stain protocol with diaminobenzidine intensification [34]. The 20 µm thick sections were deparaffinized in xylene, rehydrated in a series of alcohols, transferred to distilled water, and dried at room temperature. Then, they were transferred to the freshly prepared mixture containing 0.25 N HCl, 2% potassium ferricyanide (K3Fe(CN)6) and 8% Triton X-100 for 30 min. The sections were then rinsed in 60 mg 3,3′diaminobenzidine (DAB) in 300 mL of 0.01 M Tris-Cl (pH 7.6) with the addition of 600 µL of 30% H2O2. The incubation was carried out for three hours in complete darkness at room temperature. After incubation, the sections were rinsed in the PBS solution for 30 min. Next, the tissue was dehydrated and mounted in DPX (Merck, Darmstadt, Germany). For the negative control, endogenous peroxidase activity was determined by processing representative sections in DAB without permanent Perl reaction. All staining procedures were performed within 24 h after sectioning. Then, they were observed and photographed under the light microscope Leica DM6000B equipped with a Leica DFC 450C camera (Leica Application Suite, Germany).

### 2.9. Single Immunofluorescence Staining for Myelin Basic Protein

For immunofluorescence staining, 5 µm coronal sections were collected, deparaffinized in xylene, rehydrated in a series of alcohols, and then transferred to a citrate buffer at pH 6.0.

The sections were immersed in PBS containing 0.1% Triton X-100 at room temperature (Sigma Aldrich, St. Louis, MO, USA), blocked in 1% normal bovine serum (BSA; Sigma Aldrich, St. Louis, MO, USA) for 1 h, and incubated at 4 °C overnight with the primary antibody. Primary antibodies and dilutions were as follows: mouse anti-MBP (MilliporeMAB 382, 1:50; Merck, Darmstadt, Germany). PBS-based staining served as the negative control. The secondary antibodies were as follows: Rhodamine AffiniPure Goat Anti-Mouse IgG (Jackson Immunoresearch 115-025-003, 1:200) for 1 h (at room temperature). Rinsing in PBS containing 0.1% Triton X-100 (Sigma Aldrich, St. Louis, MO, USA) was applied between each of these steps. Scraps were counterstained with DAPI (Sigma Aldrich, St. Louis, MO, USA). The sections were embedded in glycerol (Merck, Darmstadt, Germany) and analyzed using a Leica DM6000B equipped with a Leica DFC 450C camera (Leica Application Suite, Germany).

### 2.10. Photomicrography Production

High-magnification photomicrographs of OLGs were taken with a Leica DFC 450 C digital camera controlled by the LAS program Version 4.7 (Leica Application Suite, Germany) under the Leica DM6000B microscope. As some important details were poorly seen on the row images, to improve the quality of photomicrographs, global functions (AutoLevels, Auto Contrast, and Auto Color) of Adobe Photoshop CS4, Version 11.0 (Adobe, San Jose, CA, USA) were applied, which changed the contrast and gray levels in the whole image. These functions substantially improved the readability of the photomicrographs without changing any detail.

### 2.11. Statistical Analysis

All statistical analyses were carried out using Statistica 8.0 software (StatSoft, Inc., Cracow, Poland). The unpaired two-tailed Student’s *t*-test was used. A significant difference was observed when *p* < 0.05. The results are expressed as mean ± SD. The mean coefficient of error (CE) for estimating the density of the OLGs in the NC, Hip, and CC of male and female red kangaroos was calculated according to Gundersen et al. [35]. Differences between means were analyzed using the Student’s *t*-test. *p* < 0.05 was taken as the significance criterion.

## 3. Results

### 3.1. Klüver–Barrera Staining

**Figure 2:**
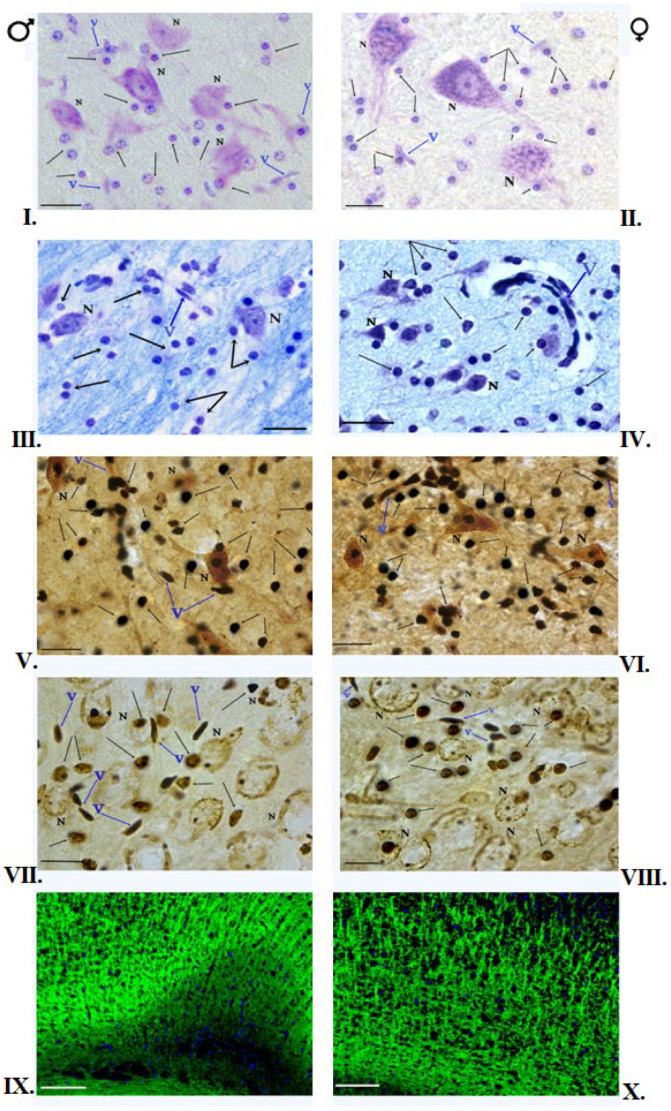
OLGs of the red kangaroo NC. (**I**,**II**)—Nissl staining (scale bar 10 μm); (**III**,**IV**)—Klüver–Barrera staining (scale bar 10 μm); (**V**,**VI**)—Glial tissue impregnation with silver salts. Arrangement of OLGs next to each other (scale bar 20 μm); (**VII**,**VIII**)—Histochemical method for detection of iron in OLGs (scale bar 10 μm); (**IX**,**X**)—Immunofluorescence staining for MBP (scale bar 50 μm). Blue nuclei stained with DAPI. Notations: ♂—male; ♀—female; N—neuron; v—blood vessel.

**Figure 3:**
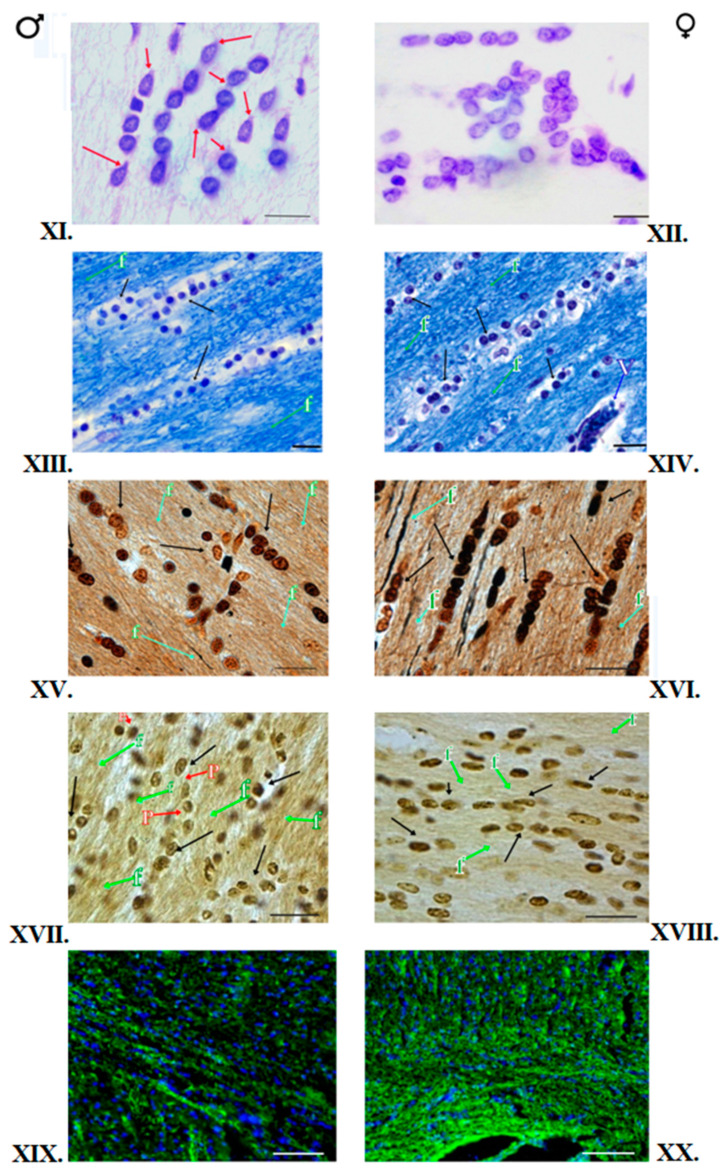
OLGs of the red kangaroo CC. (**XI**,**XII**)—Nissl staining (scale bar 10 μm, ↓—OLGs); (**XIII**,**XIV**)—Klüver–Barrera staining (scale bar 20 μm); (**XV**,**XVI**)—Glial tissue impregnation with silver salts. The traditional arrangement of OLGs in rows (scale bar 20 μm); (**XVII**,**XVIII**)—Histochemical method for detection of iron in OLGs (scale bar 20 μm); (**XIX**,**XX**)—Immunofluorescence staining for MBP (scale bar 50 μm). Blue nuclei stained with DAPI; Notations: ♂—male; ♀—female; ↓—OLGs; f—the nerve fibers; *p*—processes of OLGs; the description is in the text.

**Figure 4:**
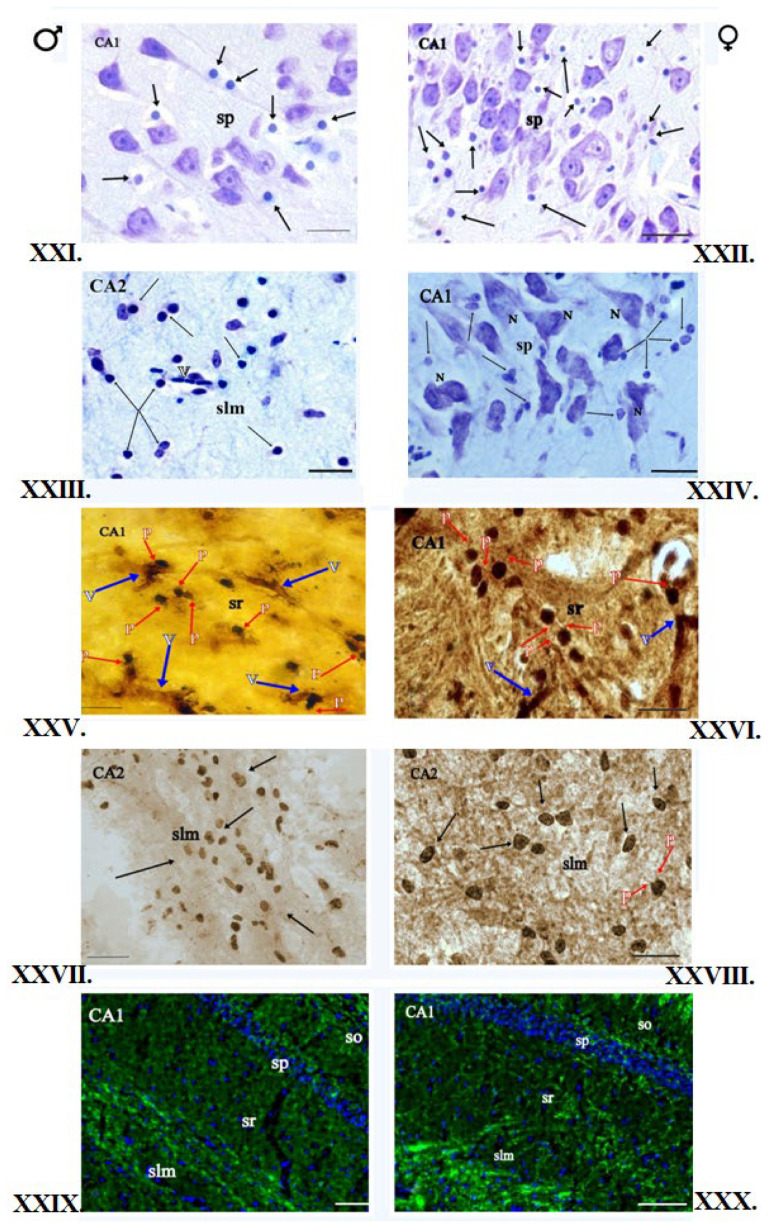
OLGs of the red kangaroo Hip. (**XXI**,**XXII**)—Nissl staining (scale bar 10 μm, OLGs—↓); (**XXIII**,**XXIV**)—Klüver–Barrera staining (scale bar 10 μm); (**XXV**,**XXVI**)—Glial tissue impregnation with silver salts (scale bar 20 μm;); (**XXVII**,**XXVIII**)—Histochemical method for the detection of iron in OLGs (scale bar 20 μm); (**XXIX**,**XXX**)—Immunofluorescence staining for MBP (scale bar 50 μm). Blue nuclei stained with DAPI; Notations: ♂—male; ♀—female; ↓—processes of OLGs; N—neuron; v—blood vessels; *p*—processes of OLGs; SO—stratum oriens, SP—stratum pyramidale, SR—stratum radiatum, SLM—stratum lacunosum-moleculare; the description is in the text.

All the slides tested have shown the proper degree of myelination. No focal or diffuse changes in myelin structure were observed (Figure 2III,IV, Figure 3XIII,XIV and Figure 4XXIII,XXIV).

### 3.2. Nissl Staining

The histological slides of the Hip in male and female kangaroos did not differ. The cytoarchitectonics of this area was visible on stained slides, and the corresponding fields and layers were connected to the location of the neurons, their shape, and distribution. Four areas specified in the literature as CA1-CA4 fields could be seen. In the fields CA1-CA4, four layers could be seen: SO, SP, SR, and SLMin in males and females. The highest number of OLGs was in the SLM layer, as well as SO and SR in the CA1-CA4 fields. OLGs are characterized by a dark round-shaped nucleus and barely visible cytoplasm. The distinction between neurons and glial cells can be seen at high magnification. In male and female brains, the OLGs are scattered in the neuropil and satellite around the neuronal soma (Figure 1). The OLGs most frequently occurred singly, but in pairs, they were accompanied by the blood vessels. They were arranged close to the neurons in the SP layer. They formed rows and accompanied the nerve fibers in the CC. Blood vessels could be seen in some places. In the NC area, the six-layer structure was visible in females and males. The individual layers of the cortex differed mainly in the content and type of nerve cells. In addition, concomitant OLGs, astrocytes, and blood vessels were visible. No morphological differences were observed in males and females in the slides (Figure 2I,II, Figure 3XI,XII and Figure 4XXI,XXII).

### 3.3. Impregnation with Silver Salts

The OLGs of spherical cellular bodies with a small number of processes were stained dark brown. In the impregnated Hip slides in the females and males, the OLGs were located mainly in blood vessels and myelinated fibers. They were present first of all in the SLM and SR layers of CA1-CA4. OLGs could sometimes be seen in neurons, mainly in the SO layer of the CA3 field. The OLGs were represented in the largest number in the layer SLM. In the NC area, cellular diversity was observed between its individual layers. The main neurons, blood vessels, single nerve fibers, and neuroglia were visible. The OLGs were unevenly distributed, mainly in deeper layers of NC, located close to the white matter. In the superficial layers of NC in males and females, OLGs occurred only singly. In females and males, OLGs were more common in the deep NC layers, mainly in layers V and VI. In the female and male CC, OLGs were arranged in rows, parallel to each other, or formed pairs, and numerous nerve fibers could be seen between them. OLGs were also found in the vicinity of blood vessels. The slides did not show differences in the morphology and location of the OLGs in females and males (Figure 2V,VI, Figure 3XV,XVI and Figure 4XXV,XXVI).

### 3.4. Histochemical Method of Detection of Iron Presence in Oligodendrocytes

In the histochemically stained slides, a brown reaction product was found in the cytoplasm, processes, and myelinated fibers of the OLGs. In the Hip, iron-positive OLGs were located mainly in sites containing myelinated fibers and blood vessels. The most abundant iron-reactive cells were detected in SLM and SR in the CA2–CA3 fields of the hippocampal, and their iron-reactive cells were round in shape, while in the SP, the iron reactivity was weak. In the CA1 field, single or in pairs, OLGs occurred most frequently in the SO or SR layers in blood vessels. In the NC, iron-positive OLGs were arranged individually, sometimes formed pairs, and were most often located in the vicinity of blood vessels or accompanied by neurons. The OLGs were found mainly in its lower layers, IV–VI, while the single ones were visible in layers II and III. The reaction product was also observed in myelin. Layers IV and V showed a slightly more intense histochemical reaction compared to the layers located under the pia mater. In the CC area, OLGs arranged in series or rows between nerve fibers at blood vessels exhibited an intense reaction product. No differences in the location and intensity of the histochemical reaction in OLGs processes and myelin sheaths surrounding the nerve fibers were observed in males and females (Figure 2VII,VIII, Figure 3XVII,XVIII and Figure 4XXVII,XXVIII).

### 3.5. Immunofluorescent Staining

The reaction product was found mainly in OLGs and myelinated nerve fibers. The fluorescent-marked cells possessed an intensely green-stained round cellular body, and rare short, fine processes could sometimes be seen. Blue cell nuclei were identified by DAPI slide staining. Fluorescent-marked OLGs in the Hip exhibited a similar pattern of location, and their intensity and distribution were consistent with those observed in the above-described staining methods. In the NC areas, in females, an even distribution of OLGs was visible in all NC layers, while in males, OLGs were located mainly in layers IIII and VI. In the CC area in males and females, the reaction product could also be seen in the nerve fibers that possess the myelin sheath (Figure 2IX,X, Figure 3XIX,XX and Figure 4XXIX,XXX).

### 3.6. Determination of Oligodendrocyte Density in Some CNS Areas

Table 1presents the data of OLG density measurements in the NC, Hip, and CC. The results were given as the density of OLG per mm3. In this study, the density of OLGs in female Hip and CC was higher than in males. Statistical analysis did not reveal significant differences between the areas of the brain studied in males and females, assuming the significance (Table 1; *p* > 0.05).

## 4. Discussion

### 4.1. Current Knowledge about OLGs

Oligodendrocyte heterogeneity was described in 1928 by del Río Hortega. He identified four types of oligodendrocytes in the central nervous system based on process numbers and orientation, soma size, and shape. However, recent studies indicate that oligodendrocytes in different regions of the brain have a wider range of morphology [1]. Many studies have investigated age-related changes in neurons and astrocytes in brains of rodents, nonhuman primates, and humans. However, very few studies have reported age-related changes in OLGs, iron reactivity, and a number of these cells in the NC, Hip, and CC. The NC is the area built mainly of neurons, blood vessels, nerve fibers, and various kinds of glial cells. The Hip mainly comprises neurons, blood vessels, nerve fibers, and different types of glial cells. However, in the CC, nerve fibers, glial cells, and blood vessels are dominant. OLGs are the most numerous cell population present in the white and gray matter of the CNS [3]. White and gray matter OLGs are characterized by similar morphology, although they differ in location toward the structural characteristics of the given brain area. In gray matter, they mainly accompany neurons, blood vessels, and other types of neuroglia, whereas in white matter, they are located in the vicinity of nerve fibers or blood vessels due to the functions performed by them [33].

### 4.2. Distribution and Role of Iron in Different Parts of CNS and Its Cells

The selective silver method enabled observation and location of OLGs in the NC, Hip, and CC in male and female red kangaroos. In the NC, OLGs were most frequently located in the lower layers, i.e., IV–VI. This was also associated with a location close to the CC rich in nerve fibers. These observations are in agreement with the studies carried out by Vaughan and Peters, as well as LeVine and Torres, who studied OLGs in the NC in mice and rats [36,37]. It was found in the NC of 25-year-old monkeys that some OLG processes combine and contain inclusion bodies of different sizes and electron densities. Some authors [38] believe that these are structural markers resulting from the aging of OLGs, which can be used in the synthesis of myelin sheaths regenerated in the process of aging. Glial tissue impregnation with silver salts revealed dark-brown OLGs, which were arranged most frequently near neurons or in the vicinity of blood vessels. Similar results were observed in mice and rats under the light microscope [37,39]. In the Hip, OLGs were topographically segregated. The differences observed in the OLGs of the hippocampus may come from the microenvironmental cues or the time at which OLG precursor cells populate the CNS. OLGs were observed most frequently in the Hip in the SLM layer of the CA1-CA4 fields. Most axons in this area come from the entorhinal cortex, which can be associated with the beginning of OLG maturation and initial myelination. The cavity is the Hip area with a large number of crossing fibers of granule and cholinergic neurons, where numerous new cells are produced after birth [40]. In the Hip, OLGs are mainly located at or close to blood vessels without concomitant astrocyte processes. These cells need a lot of iron to produce myelin. This confirms the hypothesis that they are the main CNS cells involved in iron transport from blood to the brain [6,41]. 

Moreover, the vicinity of OLGs and capillaries can explain their role in integrin maturation. This emphasizes their role in participation and preservation of the blood–brain barrier (BBB) [42]. Observation of slides stained with the silver method indicates that OLGs are not evenly distributed throughout the Hip. A similar location in this area was described for astrocytes [43]. Some studies revealed that OLGs are bound with fibers of different diameters. The morphological differences of OLGs are supposed to be dependent on the diameter of the myelinated axons or the time of their myelination [44]. In the Hip, axons come from different sources and are distributed in different ways. Entorhinal inputs are located mainly in the SLM layer of the Hip fields and in the molecular layer dentate gyrus (DG). The axons that link the pyramidal cells of the CA2 and CA3 fields reach neurons in the SR and SO. Such a location of fibers would indicate the fact that OLGs are spatially distributed in a defined Hip layer. In the OLG slides, they occurred most frequently singly between neurons in the CA2 and CA3 fields and in some places in the CA1 field. They were present between fibers from the entorhinal cortex, neurons in the SLM layer, and across the SR and SO layers. The presence of OLGs under the layer of grandmother cells can point to their contribution to the myelination axons originating from these cells [40].

Very few studies have reported age-related changes in OLGs and iron reactivity in the Hip. In the Hip, the histochemical reaction product was seen in the OLGs, and their processes and their distribution coincided with the location observed on the silver salt impregnated slides. Similar histochemical studies for the presence of iron were carried out in humans in the following areas: NC, cerebellum, olfactory bulb, striatum, Hip, and amygdala [45]. In the CC, the histochemical reaction product was visible in females’ and males’ OLGs and nerve fibers. Similar results were presented for other animal species by Francois et al. [46], Gilissen et al. [47], and Schulz et al. [48]. 

Iron-positive cells in white matter areas form lobes, the most characteristic of these areas in the human brain [45]. The iron distribution in female and male CC is consistent with the observations of Hill and Switzer [49], provided that OLGs are iron-positive cells in myelin-rich areas. Iron-enriched OLGs were also observed in birds and insects [50], suggesting that animals possessing even a small amount of myelin sheaths contain OLGs accumulating iron. As OLGs do not synthesize iron, they must acquire it [41]. Iron distribution in strongly myelinated areas is not homogeneous, and OLGs have a unique location [51]. In the developing brain, iron is present in OLGs that adhere to blood vessels.

### 4.3. Brain Cells Change Iron Requirements with Age

The functional acquisition of iron by OLGs is most probably related to their energetic metabolism. In this stage of the development of OLGs, glucose is metabolized. It is needed for the synthesis of fatty acids, myelin, and iron, which is a cofactor of key enzymes. Iron is needed for OLGs for their proper functioning, and its deficiency during development leads to hypomyelination, which was proved in studies on humans and animals [52]. Iron deficiency in rats caused a drop in MBP, PLP, lipids, and cholesterol [53]. Transferrin (TF) and ferritin (Ft) are the main proteins that supply iron in the brain [54]. TF present mainly in OLGs plays a key role during their formation, and developing OLGs possess TF-binding receptors [45,54]. In humans and adult rodents, it was proven that the expression of the TF receptor in OLG changes with age [55]. It has been suggested that there is a continuous demand for iron uptake, and its deficiency in adult individuals can result in a reduction in myelin indices [56]. It is believed that the largest demand for iron in the brain is directly after birth, e.g., in humans in the first two years after birth and in rodents in the first month after birth. Then, it drops and is maintained at a relatively low level [41]. 

Ft is a protein that stores iron, possesses a high capacity, and theoretically is capable of binding over 4500 iron atoms. Ft is composed of two chains: heavy (H) and light (L). OLGs possess two chains, but the neurons are mainly H-Ft, and the microglia are L-Ft [45,54]. Studying humans and rodents, Hulet et al. [57] showed that the presence of the H-Ft receptor in white matter is unique for OLGs, suggesting the existence of at least two alternative sources of iron supply in the brain of adult individuals [55]. Despite the apparent importance of TF for OLGs, mature OLGs in white matter contain a much lower level of the TF receptor than in other areas of the brain, for example, in the NC. Immunohistochemical studies assessing the distribution of the TF receptor in the brain exhibited strong staining of NC neurons, but staining of the TF receptors in mature OLGs was insignificant [54]. Myelin plays a very important role in learning and memory, and its degradation is a major component element in the pathogenesis of neurobiological disorders connected with cognitive functions. Perhaps the divergence of results can be explained by the age of the animal, vitality of OLGs, the maturity of the myelin sheath, and the fact that in males, there are more frequent defects in the myelin of the CNS-rich areas.

### 4.4. Metabolism Function of OLGs: Myelination

Such a specific distribution of OLGs in the Hip can suggest that these cells in this brain area perform other functions in addition to myelination. The formation of myelin sheaths in young individuals begins in all structures and then is also myelinated after birth [1]. In the CC area in male and female red kangaroos, OLGs most frequently formed characteristic rows, adhering to nerve fibers and blood vessels. They were also observed close to other glial cells. Their short processes could sometimes be seen in the slides. According to one study [58], some OLGs whose body and processes are stained a bit brighter in slides belong to OLGs which have not started myelination yet. As follows from their own investigations and those of the other authors [59], OLGs, even in adult individuals, are able to affect myelin sheaths, and the components included in OLGs or receptors specific to them can participate in the formation and enrichment of myelin. The cellular distribution of proteins associated with iron in the kangaroo brain is largely consistent with the observations made in the human, rat, and mouse brains [46]. It was proven that OLGs are the main cells containing iron; they are active in the synthesis and preservation of the myelin sheath, although the role of iron in myelination is not fully known [41]. Iron is most frequently located in the OLGs and nerve fibers, and its location is related to the metabolism of neurotransmitters [54]. Histochemically stained slides showed that in the NC cytoplasm and the processes, OLGs contained the product of reaction for the presence of iron in male and female red kangaroos.

In the deeper layer of the NC, the reaction product is located additionally between cells, which would indicate the presence of nerve fibers in this area of the NC. The authors’ own observations were similar to those made by Fukunaga et al. [60] in humans and by LeVine and Torres [37] in mice. In the NC, OLGs containing iron could be seen mainly in layers located close to the CC, and many of them were found in neurons, suggesting that they can participate in iron transport to and from neurons. The evolution of TF by OLGs in breeding [41] suggests that circumneural cells containing TF can control iron access to neurons. Iron-containing OLGs are also visible close to blood vessels, thus contributing to the control of iron transfer from plasma to the brain. The iron distribution varies, and the observation in the dog NC and CC is similar to that in the human brain [41].

### 4.5. Sexual Dimorphism in the Hypothalamus and Number of Brain Cells

For the first time, Nottebohm and Arnold [61] demonstrated the sexual dimorphism in the areas of bird brains responsible for giving voice, providing a larger number of neurons in males compared to females. NC studies using magnetic resonance showed morphological differentiation of neuron capacity in gray matter [62]. Greater gray matter capacity was found in women, but there was a larger number of neurons compared to the brain volume in the amygdala, frontal cortex, and hypothalamus [63]. Research by Cerghet et al. [64] showed a thinner myelin sheath in women and associated it with the protein composition of the myelin. The sexual dimorphism in the human and rodent hypothalamus was found to be related to reproductive functions [65]. In their studies, Hip Barrera et al. [66] found a larger number of dendrites in male neurons and proved that this area of the CNS is affected by sexual hormones that play a significant role in spatial science [67]. In dogs and rats, the studies of white matter, mainly in the CC area were confined to myelinated axons [62]. A larger number of myelinated axons was observed in males compared to females. These differences were associated with different levels of steroid hormones that affect myelination processes, indicating that the amount of myelin is correlated with the number of OLGs in both males and females [68]. This study is presumably the first to estimate the density of OLGs in male and female kangaroos using the modern unbiased morphometric technique. In the CNS areas studied, a higher density of OLGs was found in women than in men. Statistical analysis did not reveal significant differences between the NC, Hip, and CC as well as between males and females at the assumed significance of *p* < 0.05. 

An obvious aspect of the brain, particularly in humans, compared to other animal species, is its large size and a large number of neurons. The NC of mammals is subject to permanent changes in cell density, and this is one of the factors affecting changes, particularly between the layers. The studies carried out in humans, and Rhesus monkeys [11] suggest that these animals have eight times fewer neurons in the NC and about 25 times smaller number of glial cells. Some authors estimated the coefficient of the number of neurons to that of glial cells in the NC in various species, e.g., it was 0.6/1 in humans, 1.7/1 in monkeys [11], and 0.45/1 in the miniature pigs Göttingen Minipig [69]. The glial cells adapt to the population of neurons in various areas of the brain. The tendency toward a larger glial cell number than that of neurons is found in animals with a larger brain mass, which can be associated with providing metabolic support for nerve cells [70]. The density of neurons in the NC decreases with increasing brain size [71]; however, the density of glial cells, which was believed to be independent of brain mass, was seen to be on a stable level [11].

All cellular elements of the NC have layered arrangements, including projection neurons, interneurons, and OLGs, except for blood vessels, astrocytes, and microglia. Keuker et al. [72] studied the Hip of Rhesus monkeys, though only to determine the number of neurons, and found that in humans, about four times more neurons occur in the CA1 field and about two times more in the CA2–CA3 fields. In rats, areas rich in myelinated fibers showed a significant drop in the number of OLGs with age [73]. A smaller number of OLGs in older animals can depend on the change in nerve fibers. One reason for this is the higher risk of ischemia and hypoxia in the brain, particularly in the area of white matter [74] because OLGs are very susceptible to such damage [75]. Some researchers suggest that changes in blood vessels can be one of the reasons for the change in OLG density in adult individuals. Furthermore, OLGs are sensitive to oxidative stress, which contributes to the degeneration of these glial cells, which causes ischemia damage in the brain and demyelination diseases [73]. A study by Reyes-Haro et al. [76] on the CC in rats showed the difference in the regional density of glial cells, which could result from changes in CC sizes and the number of axons in this area. The participation of OLGs in the myelination process and the preservation of myelin sheaths is very complicated. The formation of OLGs occurs during life, contributing mainly to the formation of myelin sheaths and the restructuring of myelin, which can play a role in memory.

### 4.6. What This Paper Adds

Contemporary studies provide more and more information about the function of OLGs. Present knowledge on the function of OLGs focuses mainly on myelination processes in young individuals. However, knowledge about their role after myelin sheath formation is poor. The research results indicate that the OLGs are not fully understood with respect to the morphological and functional aspects. Although the participation of OLGs in the myelination process in young individuals is well documented, the presence of iron in these cells is not yet clear. These data lead to new questions concerning the development, organization, and involvement of these cells in metabolic processes at the genetic and immunological levels. Furthermore, the observation that a large number of OLGs have a close relationship with capillaries suggests an active role in the BBB. Better knowledge of these cells can prove to be a key element in the development of new therapeutic strategies for many neurodegenerative diseases in which OLGs are included.

## 5. Conclusions

In conclusion, using a powerful morphometric approach, we were able for the first time to estimate the density of OLGs in NC, Hip, and CC in male and female red kangaroos. The research results indicate that the location of OLGs in nerve fibers, blood vessels, and neurons prove that they are equally engaged in metabolic processes of accompanying neurons independent of sex and age of individuals. These results could be used as a morphological basis for understanding the biology of OLGs.

## Figures and Tables

**Table 1:** The density of OLGs/mm^3^ in the selected brain areas of the red kangaroo.

Brain Areas	NC		Hip		CC	
	♂♀		♂♀		♂♀	
MEAN±	116,729.94	98,035.95	119,204.78	127,202,36	404,543.75	431,927.92
MEAN ± SD	24,456.56	7765.22	30,313.19	37,355.54	70,017.60	66,301.72

♂, male; ♀, female; NC, neocortex; Hip, hippocampus; CC, corpus callosum; SD, standard deviation.

## Data Availability

The data used to support the findings of this study are included in the article.
